# Emotion regulation versus impulsivity in alcohol and cannabis dependence

**DOI:** 10.6026/973206300221647

**Published:** 2026-03-31

**Authors:** Devesh Kumar Vyas, Soram Nganbaren, Sandeep Kumar Jain, Vishnu Kumar Gupta, Rupesh Sahu

**Affiliations:** 1 Department of Psychiatry, SRVS Medical College Shivpuri, Madhya Pradesh, India; 2 Department of Medicine, Chhindwara Institute of Medical Sciences, Chhindwara, Madhya Pradesh, India; 3 Department of Community Medicine, SRVS Medical College Shivpuri, Madhya Pradesh, India; 4 Department Community Medicine, Chhindwara Institute of Medical Sciences, Chhindwara, Madhya Pradesh, India

**Keywords:** Alcohol dependence, cannabis dependence, emotion regulation, impulsivity, barratt impulsiveness Scale (BIS-30), emotion regulation questionnaire (ERQ)

## Abstract

Emotion dysregulation and impulsivity are key psychological factors contributing to substance dependence and relapse. Therefore, it is of interest to assess and compare emotion regulation and impulsivity in patients with alcohol dependence syndrome and cannabis dependence syndrome. A hospital-based cross-sectional study was conducted with 64 male patients, using the Emotion Regulation Questionnaire (ERQ) and Barratt Impulsiveness Scale (BIS-30) for assessment. Results showed alcohol-dependent patients had poorer emotion regulation and higher impulsivity than cannabis-dependent patients. This research advances knowledge by highlighting the greater severity of emotion dysregulation and impulsivity in alcohol dependence, emphasizing the need for targeted psychological interventions.

## Background:

Substance dependence is a chronic relapsing condition defined by compulsive substance use despite negative consequences, which causes considerable psychological, social and bodily impairment [1]. Alcohol and cannabis are the most commonly used psychoactive substances worldwide, contributing significantly to the global disease burden [2]. According to India's National Survey on Extent and Pattern of Substance Use, alcohol is the most commonly used substance and cannabis is the most widely used illegal drug, with a considerable number of users matching dependence criteria [3]. Emotion regulation refers to the methods by which people control their emotions, when they experience them and how they express them [4]. Deficits in emotion regulation are significantly linked to substance dependence, as people frequently use substances as maladaptive coping techniques to deal with uncomfortable feelings [5]. Gross's process model identifies cognitive reappraisal and expressive suppression as crucial emotion regulation techniques, with maladaptive patterns predicting poor mental health outcomes and relapse [6]. Impulsivity is described as the tendency to behave without enough consideration and is seen as a key trait of addictive illnesses [7]. Elevated impulsivity makes people more likely to start using drugs, develop addiction and relapse. According to neurobiological hypotheses, decrease impulse control in substance use disorders results from dysfunction in the prefrontal cortex and reward pathways [8]. Alcohol dependence is associated with significant emotional dysregulation, increased stress reactivity and impulsivity, all of which contribute to risky behavior and poor treatment outcomes [9]. Cannabis dependence has been associated to poor emotion processing and executive dysfunction, however the pattern and severity differs from alcohol dependence [10]. Therefore, it is of interest to test and compare emotion regulation and impulsivity in patients with alcohol and cannabis dependence syndrome attending a tertiary care hospital.

## Methodology:

The study was conducted in the Department of Psychiatry at Chirayu Medical College and Hospital, Bhopal, which provided access to a diverse patient population diagnosed with alcohol and cannabis dependence syndrome. A total of 64 male patients aged 18-50 years (32 with alcohol dependence and 32 with cannabis dependence) were diagnosed according to ICD-10 criteria. The study lasted for 18 months. Inclusion criteria were male patients aged between 18 and 50 years, diagnosed with alcohol or cannabis dependence syndrome as per ICD-10 and willing to provide written informed consent. Exclusion criteria included patients with multiple drug dependence, pre-existing psychiatric illnesses like schizophrenia or bipolar disorder, chronic medical conditions affecting psychiatric health, alcoholic hallucinosis, a history of head injury, or those experiencing acute withdrawal symptoms. Statistical analysis was conducted with the hypothesis that there would be significant differences in emotion regulation and impulsivity between the two groups. Descriptive statistics summarized demographic and clinical data, while t-tests were used to compare emotion regulation and impulsivity scores. Associations between demographic factors and study outcomes were analyzed using chi-square tests or correlation analyses. Data collected on paper were entered into MS Excel and imported into Stata software version 17.0 for all statistical and graphical analyses, with a significance level set at p < 0.05.

## Results:

A total of 64 male participants aged between 18 and 50 years were enrolled in the study, with 32 individuals in each diagnostic group (alcohol dependence and cannabis dependence), selected through non-probability convenience sampling method. All participants met the ICD-10 diagnostic criteria for their respective substance use disorders and provided written informed consent prior to enrolment. The study was conducted over an 18-month period and adhered to all ethical and methodological protocols approved by the Institutional Ethics Committee. A comparative analysis was performed to examine differences in emotion regulation and impulsivity between the two groups using appropriate statistical methods. Table 1 (see PDF) summarizes the socio-demographic characteristics of the study participants. Alcohol-dependent patients were predominantly in the 31-40 year age group, while cannabis-dependent patients were younger, with most in the 21-30 year age group. Senior secondary education was most common in the alcohol dependence group, whereas college education predominated among cannabis- dependent patients. Higher proportions of alcohol-dependent patients were from rural areas and were married, while cannabis-dependent patients were mainly urban residents and unmarried. According to Kuppuswamy's socioeconomic classification, alcohol-dependent patients mostly belonged to the upper-middle class, whereas cannabis-dependent patients were commonly from the upper-lower class. Table 2 (see PDF) compares emotion regulation between the two groups. Alcohol-dependent patients had lower cognitive reappraisal scores (26.1 ± 3.8) and higher expressive suppression scores (20.7 ± 2.71) compared to cannabis-dependent patients (27.2 ± 3.86 and 19.5 ± 2.53 respectively), although these differences were not statistically significant. The total ERQ score was almost similar in both groups (46.8 ± 4.61 versus 46.7 ± 4.78; p = 0.915). Table 3 (see PDF) shows a significant difference in impulsivity between the two groups. Alcohol-dependent patients scored significantly higher on attention impulsivity (29.5 ± 2.38 versus 23.7 ± 2.48; p = 0.002), behavioural impulsivity (29.6 ± 4.17 versus 24.8 ± 3.56; p = 0.008) and non-planning impulsivity (30.4 ± 5.46 versus 26.5 ± 4.6; p = 0.0027) compared to cannabis-dependent patients. The total impulsivity score was also significantly higher in the alcohol dependence group (89.5 ± 7.65) than in the cannabis dependence group (75 ± 6.96; p < 0.0001).

## Discussion:

The study found that alcohol-dependent individuals have poorer emotion management and more impulsivity than cannabis-dependent patients. Similar findings have been observed in studies linking alcohol dependence to increased emotional dysregulation and relapse vulnerability [11]. Longer periods of alcohol dependence were associated with increased suppression and impulsivity, similar with previous research indicating that chronic alcohol use impairs prefrontal inhibitory function [12]. Cannabis-dependent individuals, although being younger and more educated, demonstrated severe abnormalities in emotion regulation and impulsivity, indicating that cannabis affects executive functioning and emotional processing [13]. According to the ERQ findings, weaker cognitive reappraisal and higher suppression in alcohol dependence predict worse coping and a higher likelihood of relapse, as previously reported [14]. BIS-30 data highlight impulsivity as a significant predictor of relapse and treatment non-adherence [14]. These findings emphasize the need for including emotion regulation and impulse control training into de-addiction programs. Interventions like CBT, DBT and mindfulness-based relapse prevention have been effective in addressing these psychological domains. The importance of addressing emotion regulation and impulse control in addiction treatment is clear. Our study, along with the findings of Mostafa *et al.* (2026) [15] and Nganbaren *et al.* (2025) [16], underscores the need for incorporating psychological interventions such as Cognitive Behavioral Therapy (CBT), Dialectical Behavior Therapy (DBT), and mindfulness-based relapse prevention to target these core issues. These therapies have shown to be effective in improving emotional regulation, impulse control and relapse prevention in individuals with substance use disorders.

## Conclusion:

Emotion dysregulation and impulsivity are key psychological deficiencies in both alcohol and cannabis dependence, while alcohol dependence is more severe. Early detection and tailored psychological therapies aimed at emotion regulation and impulsivity may improve treatment success and reduce relapse rates.
